# Metformin mediates cardioprotection against aging‐induced ischemic necroptosis

**DOI:** 10.1111/acel.13096

**Published:** 2020-01-14

**Authors:** Chen Li, Nan Mu, Chunhu Gu, Manling Liu, Zheng Yang, Yue Yin, Mai Chen, Yishi Wang, Yuehu Han, Lu Yu, Heng Ma

**Affiliations:** ^1^ Department of Physiology and Pathophysiology Fourth Military Medical University Xi'an China; ^2^ Department of Cardiovascular Surgery Xijing Hospital Fourth Military Medical University Xi'an China; ^3^ Department of Cardiovascular Medicine Xijing Hospital Fourth Military Medical University Xi'an China; ^4^ Department of Pathology Xijing Hospital Fourth Military Medical University Xi'an China

**Keywords:** aging, autophagy defect, cardioprotection, ischemia/reperfusion injury, metformin, myocardial necroptosis

## Abstract

Necroptosis is crucially involved in severe cardiac pathological conditions. However, whether necroptosis contributes to age‐related intolerance to ischemia/reperfusion (I/R) injury remains elusive. In addition, metformin as a potential anti‐aging related injury drug, how it interacts with myocardial necroptosis is not yet clear. Male C57BL/6 mice at 3–4‐ (young) and 22–24 months of age (aged) and RIPK3‐deficient (*Ripk*3^−/−^) mice were used to investigate aging‐related I/R injury in vivo. Metformin (125 μg/kg, i.p.), necrostatin‐1 (3.5 mg/kg), and adenovirus vector encoding p62‐shRNAs (Ad‐sh‐p62) were used to treat aging mice. I/R‐induced myocardial necroptosis was exaggerated in aged mice, which correlated with autophagy defects characterized by p62 accumulation in aged hearts or aged human myocardium. Functionally, blocking autophagic flux promoted H/R‐evoked cardiomyocyte necroptosis in vitro. We further revealed that p62 forms a complex with RIP1‐RIP3 (necrosome) and promotes the binding of RIP1 and RIP3. In mice, necrostatin‐1 treatment (a RIP1 inhibitor), RIP3 deficiency, and cardiac p62 knockdown in vivo demonstrated that p62‐RIP1‐RIP3‐dependent myocardial necroptosis contributes to aging‐related myocardial vulnerability to I/R injury. Notably, metformin treatment disrupted p62‐RIP1‐RIP3 complexes and effectively repressed I/R‐induced necroptosis in aged hearts, ultimately reducing mortality in this model. These findings highlight previously unknown mechanisms of aging‐related myocardial ischemic vulnerability: p62‐necrosome‐dependent necroptosis. Metformin acts as a cardioprotective agent that inhibits this unfavorable chain mechanism of aging‐related I/R susceptibility.

## Graphical abstract

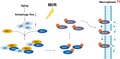

## 1 INTRODUCTION

Aging hearts work on the edge of disease (Dai, Chen, Johnson, Szeto, & Rabinovitch, 2012; Xu et al., 2018). Aging is associated not only with a progressive decline in physical function, but also with a significant increase in the risk of many disabilities and even death. Intrinsic functional declines induced by aging make the heart more susceptible to ischemia/reperfusion (I/R) injury and contribute to increased cardiovascular mortality and morbidity in the elderly (Ma et al., 2010; Ren & Zhang, 2018; Xing, Sun, Wang, Gao, & Ma, 2016). Identifying the molecular basis of aging‐related ischemic vulnerability is not only scientifically important but may also reveal new therapeutic targets.

During the last several years, scientific studies on aging have demonstrated that metformin has beneficial effects on aging‐related damage in cellular and animal models and even cardioprotection against myocardial I/R injury (Calvert et al., 2008). But, dispute still exists. For instance, metformin protects the heart from cardiac hypertrophy induced by aging or other stresses (Tang et al., 2017). Nevertheless, Techyran et al. reported that administration of metformin intravascularly before reperfusion to achieve high intracoronary plasma levels in a porcine model (3–4 months of age) failed to reduce infarct size (Techiryan, Weil, Palka, & Canty, 2018). Hence, the exact molecular mechanisms of metformin‐mediated cardioprotection in aging hearts remain unclear.

The loss of terminally differentiated cardiomyocytes after injury is an important pathogenic factor in the development of heart failure. Necroptosis is a programmed cell death process that is orchestrated by a complex set of proteins involving receptor‐interacting protein kinases 1 and 3 (RIP1, RIP3) and mixed lineage kinase domain‐like protein (MLKL). Mechanistically, RIP1 and RIP3 form the “necrosome” (Cho et al., 2009) and subsequently phosphorylate MLKL (Sun et al., 2012), MLKL then translocates to the cell membrane and induces cell membrane rupture and necroptosis (Cai et al., 2014). Recent information has indicated the existence and importance of necroptosis in myocardial I/R injury (Zhang et al., 2016). However, the role of necroptosis in the increased ischemic vulnerability observed with age has yet to be demonstrated. Furthermore, the molecular mechanisms of age regulating myocardial necroptosis remain unclear.

Aging results in progressive deteriorations in cardiomyocyte intrinsic function and is a subclinical basis for cardiovascular diseases. Autophagy and autophagic flux are generally decreased in aging hearts (Shirakabe, Ikeda, Sciarretta, Zablocki, & Sadoshima, 2016). The accumulation of biological macromolecules and abnormal organelles during cardiac aging can lead to intracellular homeostasis imbalance and ultimately cardiomyocyte death (Nah, Fernandez, Kitsis, Levine, & Sadoshima, 2016). This raises the possibility that significant autophagic component accumulation can directly affect cell survival or death signals through nonautophagic functions. p62/SQSTM1 is a protein with many biological functions; in particular, it plays an important role in abnormal protein degradation. p62 is considered a "bridge" between autophagy and its substrates, and it is an important mediator of functional autophagic flux. p62 accumulation is believed to cause organ dysfunction, such as in the liver and brain (Hara et al., 2016; Takamura et al., 2011). Recent data have also shown that p62 can serve as a scaffold to modulate the mode of programmed cell death in cancer cells (Goodall et al., 2016) or neurons (Liu et al., 2018). However, in aged hearts, definitive in vivo evidence that p62 regulates stress‐induced cardiomyocyte death is less well established. The relationship between necroptosis and autophagy during cardiac aging is incomplete, and such crosstalk mechanisms in pathophysiological settings (i.e., I/R injury) have never been determined in vivo in aging individuals.

Here, we demonstrated that metformin suppresses p62‐RIP1‐RIP3 complex‐driven necroptosis in aged hearts. Aging‐associated autophagic defects contribute to the increased myocardial necroptosis induced by I/R. We found that accumulated p62 forms a complex with RIP1‐RIP3 and promotes the binding of RIP1 and RIP3 and subsequent MLKL phosphorylation, resulting in necroptosis in aged I/R hearts. Metformin treatment can restore autophagy and disrupt the p62‐RIP1‐RIP3 complexes, effectively reducing I/R‐induced necroptosis in aging hearts. Impaired autophagosome clearance is one cornerstone in I/R‐induced myocardial necroptosis.

## 2 RESULTS

### 2.1 Autophagic defects and necrosis enhancement with aging

**Figure 1:**
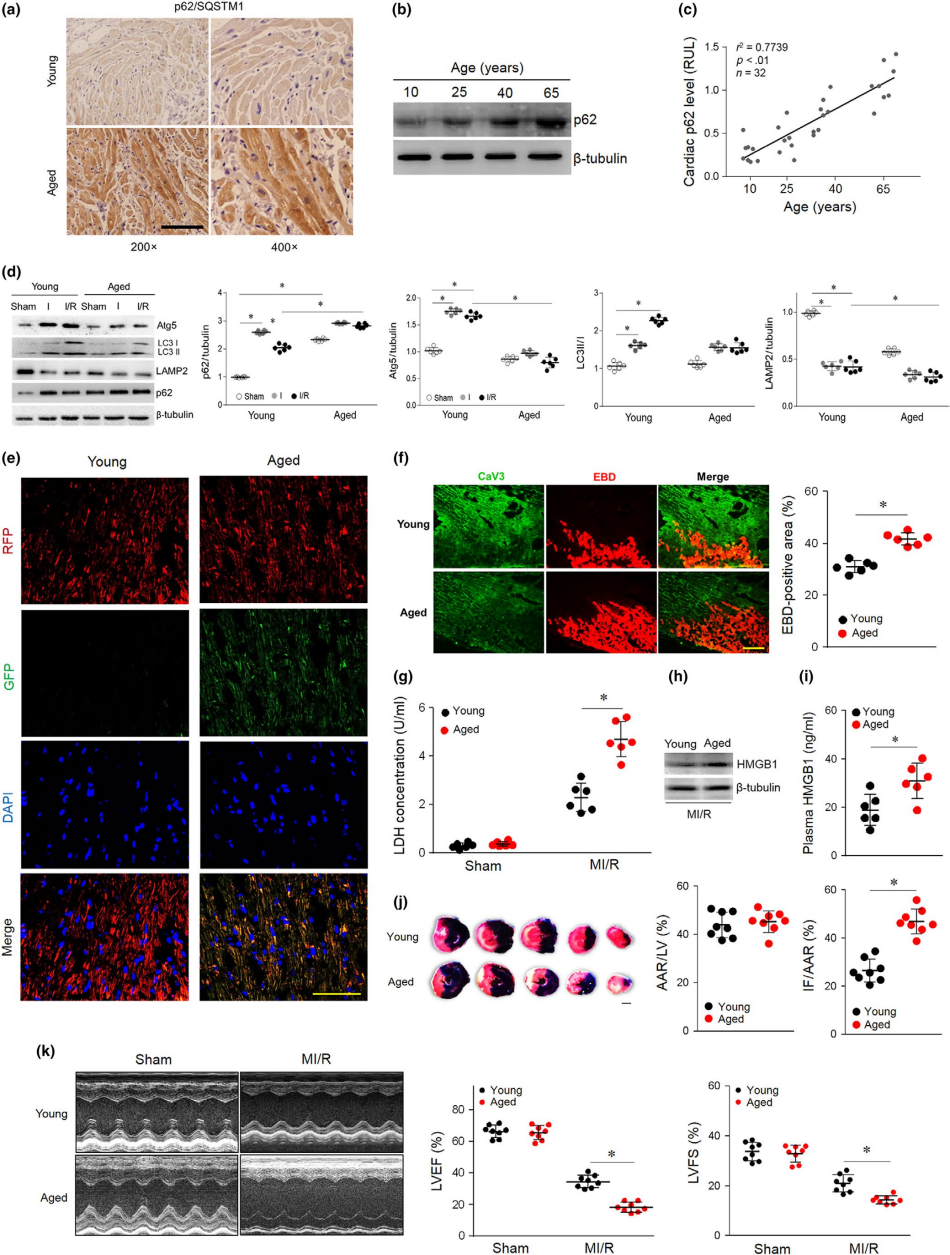
Impaired autophagic flux and enhanced necrosis. (a) Representative images of myocardial p62 immunohistochemical staining in young (10 years) and aged (65 years) human samples. (b) Representative Western blot for p62 protein levels in human myocardium samples from patients of different ages. (c) Linear regression analysis revealed a tendency for myocardial p62 to be positively related to age in the studied population. (d) Young and aged hearts were subjected to I/R (ischemia for 30 min and reperfusion for 120 min). Western blot analyses and quantification data of cardiac Atg5, LC3 II/I, LAMP2, and p62 levels. (e) Young and aged hearts were transfected with AAV9‐RFP‐GFP‐LC3 4 weeks prior to I/R surgery. Myocardial RFP‐GFP‐LC3 was stained and imaged by immunofluorescence. (f) Myocardial necroptosis was assessed by EBD uptake (red) in young and aged hearts subjected to ischemia (30 min) and reperfusion (240 min). (g) Serum LDH concentrations in each group. (h and i) Cardiac and plasma levels of HMGB1 in each group. (j) Area at risk (AAR) and infarct size (IF) in hearts subjected to I/R. (k) Average data for left ventricle ejection fraction (LVEF) and fractional shortening (LVFS) were assessed by echocardiography in young and aged mice subjected to sham or I/R injury (30 min of ischemia and 4 weeks of reperfusion). Scale bar = 20 μm. The values are the means ± *SEM*, *n* = 6 or 8 per group, *p < .05 versus the indicated groups

To investigate the correlation of cardiac p62 levels with age, p62 immunostaining was performed in human myocardial samples from young (10 years) and aged (65 years) patient hearts. The p62 levels were significantly higher in aged hearts than in young hearts (Figure 1a), whereas a positive correlation was observed between patient age and myocardial p62 protein level (age range 7–69 years, *n* = 32, *r* ^2^ = .77, *p* < .01) (Figure 1b,c). These results indicated age‐related p62 accumulation in human hearts.

To determine baseline and MI/R autophagosome clearance, we evaluated the state of the autophagy/lysosome system in vivo in young and aged mouse hearts. Young and aged mice demonstrated a normal baseline cardiac phenotype and function (Table S1). Young and aged mice were subjected to 30 min of ischemia followed by 4 hr of reperfusion. A sham operation group was used for parallel observation (Figure S1). Consistent with the clinical sample results, the p62 levels were significantly higher in the aged hearts than in the young hearts in the sham‐operated groups (Figure 1d). Intriguingly, in the young hearts, Atg5 and LC3‐II levels were higher, and lysosome formation (LAMP2) was lower during ischemia and reperfusion than during the sham operation (Figure 1d). p62 was increased in the ischemic period and degraded in the reperfusion period, which indicated that autophagy flux and autophagosome clearance were efficient in the young hearts. However, Atg5, LC3‐II, and LAMP2 levels were lower in the aged hearts than in the young hearts during ischemia and reperfusion. More importantly, p62 was increased during ischemia in the aged groups and accumulated significantly in the reperfusion period (Figure 1d), suggesting that autophagosome clearance was impaired in aged myocardial I/R injury. To observe the dynamic changes of autophagy flux in vivo, hearts were injected with AAV9‐mRFP‐GFP‐LC3 and subjected to I/R operation 4 weeks later. When the mRFP‐GFP‐LC3 fusion protein fuses with acidic lysosomes, only red fluorescence is observed, and GFP is quenched. Yellow fluorescence is observed when autophagosome clearance is impaired. Fluorescence in vivo results also indicated that autophagosome clearance was crippled in aged MI/R hearts (Figure 1e). Thus, these data demonstrate that impaired autophagosome clearance results in p62 accumulation in aged MI/R hearts.

We also found that MI/R‐induced cardiac necrosis was markedly enhanced in aged mice, as evidenced by enhanced Evans blue dye (EBD) penetration (Figure 1f) and lactate dehydrogenase (LDH) release (Figure 1g) in aged hearts. In addition, high‐mobility group box‐1 (HMGB1) is a nuclear factor released by necrotic cells during MI/R (Andrassy et al., 2008). Compared with young mice, MI/R injury resulted in significantly higher HMGB1 cardiac levels (Figure 1h) and plasma release (Figure 1i) in aged mice. Specifically, aged mouse hearts had larger myocardial infarct sizes (Figure 1j) and lower cardiac contractile function (Figure 1k) than young mouse hearts. Together, the in vivo data indicate that autophagic defects increased p62 accumulation and enhanced I/R‐induced myocardial necrosis in aging myocardium.

### 2.2 Blocking autophagic flux promotes H/R‐induced cardiomyocyte necroptosis

**Figure 2:**
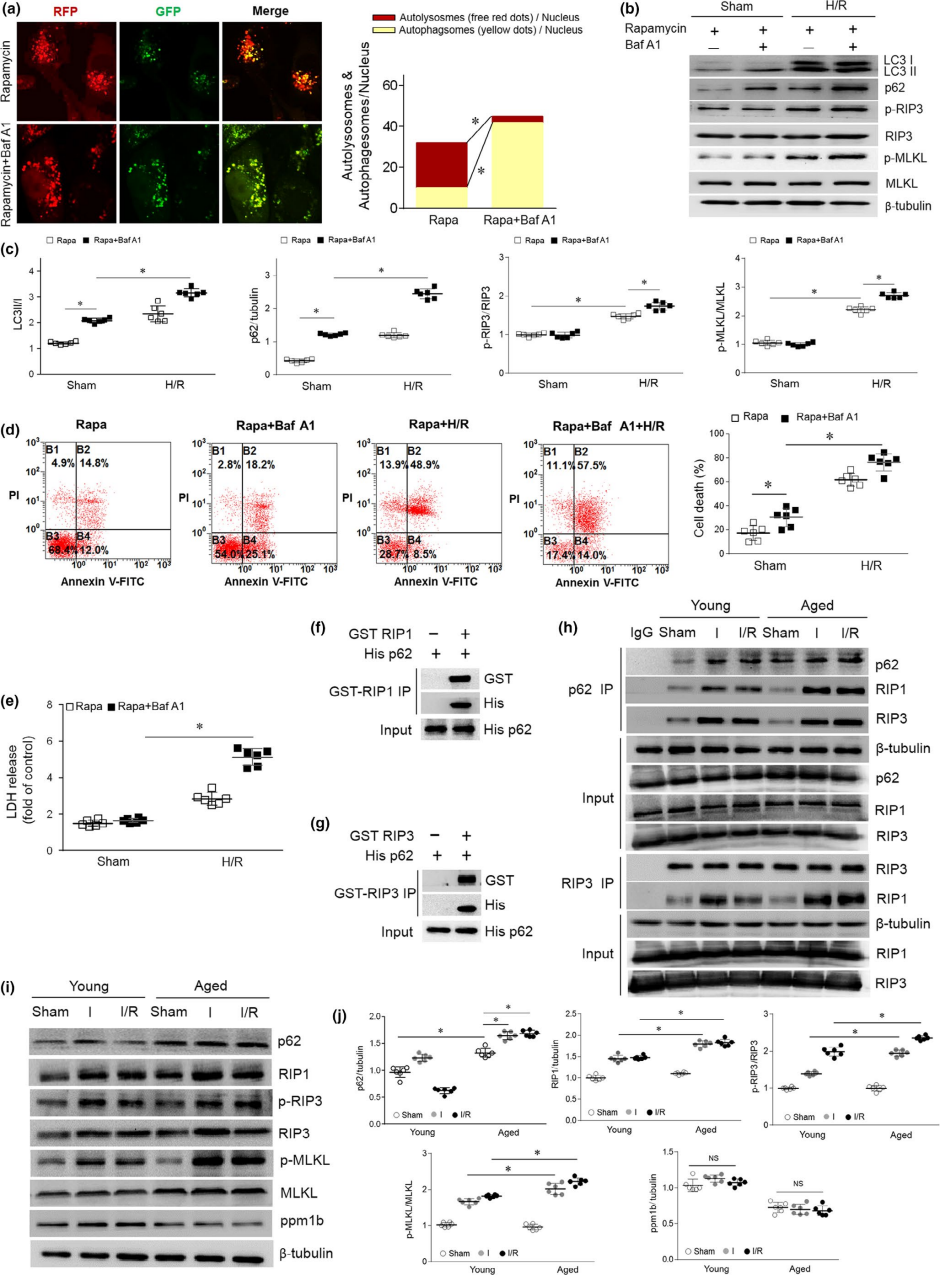
Autophagy blockade enhanced necrosis and p62‐RIP1‐RIP3 interaction. (a) Cultured primary cardiomyocytes were subjected to rapamycin (100 nM) or rapamycin plus bafilomycin A1 (100 nM) treatment. Representative images of fluorescent LC3 puncta after mRFP‐GFP‐LC3 adenovirus transduction (24 hr). The mean number of autophagosomes is represented by the yellow puncta in the merged images, and the autolysosomes are represented by the red puncta in the merged images for each cell. (b and c) Representative Western blot and quantification data for LC3 I/II, p62, p‐RIP3/RIP3, and p‐MLKL/MLKL in cardiomyocytes treated with rapamycin or rapamycin plus bafilomycin A1 under sham or H/R conditions. D, Cardiomyocytes were incubated with Annexin V‐FITC and then subjected to flow cytometry to evaluate cell death. (e) Supernatant LDH levels were detected by LDH assay. (f) and (g) RIP3‐p62 and RIP1‐p62 combinations were detected by GST pull‐down assays. (h) Co‐immunoprecipitation of the p62‐RIP1‐RIP3 complex and the RIP1‐RIP3 complex in young and aged hearts subjected to sham, ischemia or I/R. (i and j) Representative Western blot and quantification data for p62, RIP1, p‐RIP3/RIP3, p‐MLKL/MLKL and ppm1b in young and aged hearts subjected to sham, ischemia or I/R. Scale bar = 20 μm. The values are the means ± *SEM*, *n* = 6 per group, *p < .05 versus the indicated groups

We next evaluated the functional relationship between autophagy inhibition and necroptosis. Primary cultured cardiomyocytes were labeled with tandem fluorescent‐tagged LC3 (Ad‐tf‐LC3). We performed an LC3 flux analysis using rapamycin with or without bafilomycin A1 treatment; this method provides a simultaneous readout of autophagosome formation. Autophagosome clearance was blocked by bafilomycin A1 and was confirmed by immunofluorescence (Figure 2a). Western blot analyses revealed increased p62 abundance in the rapamycin plus bafilomycin A1‐treated cardiomyocytes. In addition to this finding, hypoxia‐reoxygenation (H/R), a condition mimicking in vivo I/R in cultured cardiomyocytes, further increased p62 levels (Figure 2b,c). Consistent with our hypothesis, in the case of p62 accumulation, H/R‐induced RIP3 and MLKL phosphorylation was markedly enhanced (Figure 2b,c). Moreover, inhibiting autophagic flux by bafilomycin A1 profoundly increased H/R‐induced myocardial necrosis, as indicated by cell death according to positive staining for both annexin V and propidium iodide using the FACS technique (Figure 2d) and increased LDH concentrations (Figure 2e). These data further supporting the concept that blocking autophagy positively regulates H/R‐induced necroptosis in cardiomyocytes.

### 2.3 p62 binds the RIP1‐RIP3 complex

We next sought to identify the missing link between autophagy and necroptosis. RIP1 forms a complex with RIP3 that induces necroptosis (Luedde et al., 2014). Nonetheless, whether p62 affects the interaction of RIP1 and RIP3 in aging hearts is unclear. First, we tested the binding using a GST pull‐down assay and found that there is a direct binding relationship between p62 and RIP3 and between p62 and RIP1 (Figure 2f,g). Based on its novelty and potential biological importance, we next studied the interaction of p62 and the RIP1‐RIP3 complex in vivo. We evaluated the co‐immunoprecipitation of p62 and RIP1‐RIP3 in young and aged hearts with or without 30 min of ischemia followed by 4 hr of reperfusion. RIP1‐RIP3 complex (necrosome) formation induced by I/R was significantly increased in aged hearts (Figure 2h), consistent with the enhanced necrosis in aged myocardium. Notably, Co‐IP assays revealed a physical interaction between p62 and the necrosome, which was markedly enhanced by ischemia and reperfusion (Figure 2h). p62‐RIP1‐RIP3 binding was higher in aged hearts than in young hearts (Figure 2h). Meanwhile, p62 levels and RIP1, p‐RIP3, and p‐MLKL expression were increased by I/R and aggravated in aged hearts (Figure 2i,j). Compared with that in young hearts, necroptosis signaling was activated during I/R and aggravated in aged hearts, as indicated by increased RIP3 and MLKL phosphorylation. No significant change in RIP3 phosphatase ppm1b levels during I/R excludes the effect of dephosphorylation (Figure 2i,J). MLKL phosphorylation activation and membrane translocation were reported as the final steps in necroptosis (Cai et al., 2014). Indeed, fluorescence in vivo results revealed that I/R distinctly increased p‐MLKL membrane translocation, which was more pronounced in senescent hearts (Figure S2). These new in vivo data suggest that p62 accumulation in aging hearts might promote necroptosis signals during I/R. The interaction of the p62‐RIP1‐RIP3 complex may strengthen necroptosis in the aging myocardium.

### 2.4 Suppressing the p62‐RIP1‐RIP3 complex reduces necroptosis

**Figure 3:**
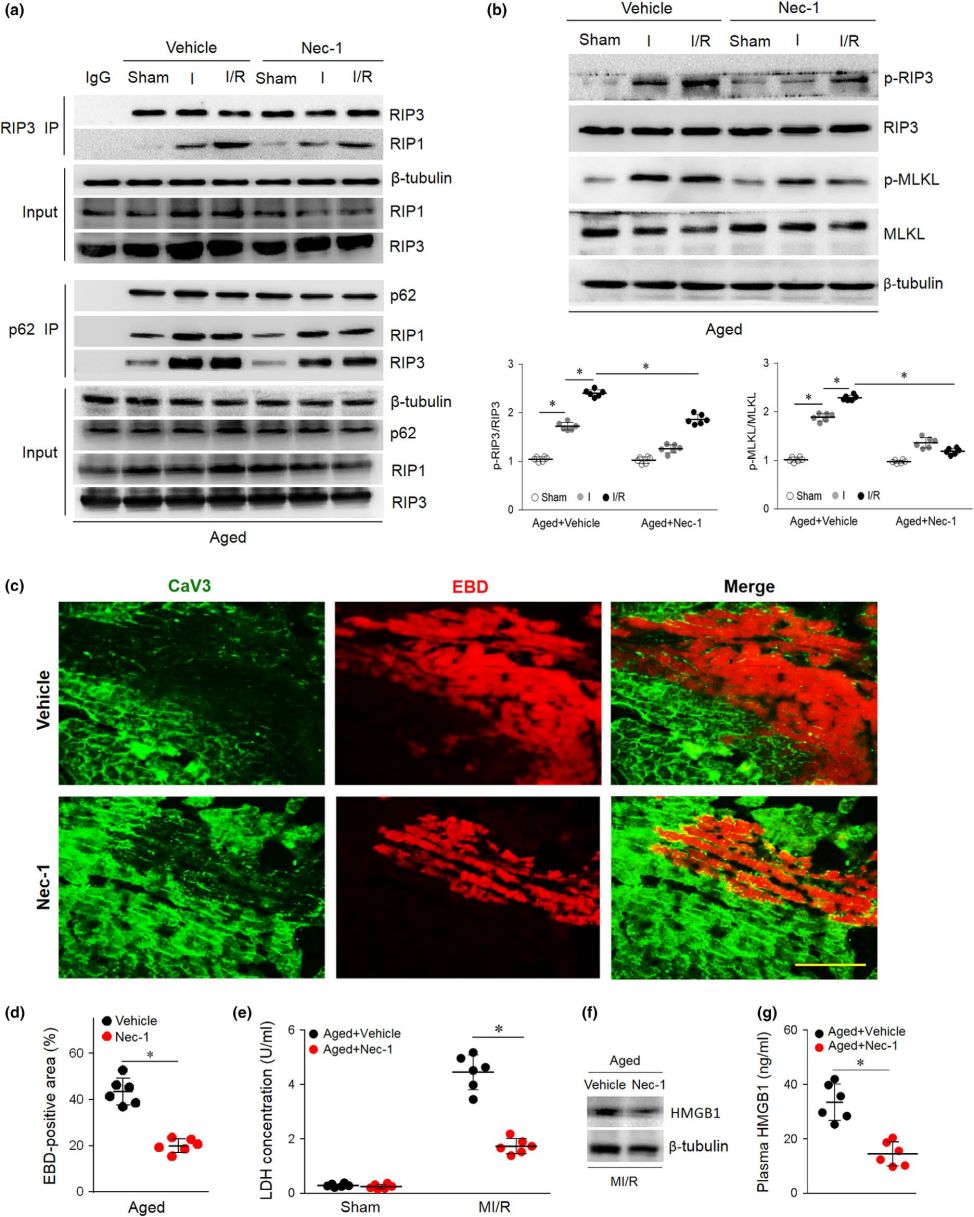
Necroptosis participates in aging‐related MI/R injury enhancement. (a) Aged mice were injected with Nec‐1 (3.5 mg/kg) or vehicle as a control via their tail vein 5 min prior to ischemia. The p62‐RIP1‐RIP3 complex and the RIP1‐RIP3 complex were examined by co‐immunoprecipitation. (b) Myocardial p62, p‐RIP3/RIP3, and p‐MLKL/MLKL were detected by immunoblots. (c and d) Representative photomicrographs and averaged data from the images of myocardial EBD uptake and viable cardiomyocytes labeled with caveolin 3 (CaV3) antibody in aged mice subjected to I/R injury with or without Nec‐1 treatment. (e) Serum LDH concentration in each group. (f and g) Representative Western blot and averaged data for cardiac or plasma HMGB1 levels in aged mice subjected to I/R injury with or without Nec‐1 treatment. Scale bar = 20 μm. The values are the means ± *SEM*, *n* = 6 per group, *p < .05 versus the indicated groups

We observed the effects disrupting the p62‐RIP1‐RIP3 complex on aged myocardium. Nec‐1 is a pharmacological inhibitor of RIP1 that blocks the RIP1‐RIP3 interaction and inhibits necroptosis (Degterev et al., 2005). The aged mice were subjected to Nec‐1 treatment or vehicle control treatment followed by MI/R operation. As expected, Nec‐1 treatment markedly inhibited the RIP1‐RIP3 interaction in aged hearts and reduced I/R‐induced myocardial p62‐RIP1‐RIP3 complex formation (Figure 3a). Nec‐1 treatment reduced the MI/R‐induced RIP3 and MLKL phosphorylation in aged hearts (Figure 3b) and ultimately induced resistance to I/R‐induced myocardial necrosis, as evidenced by reductions in EBD‐positive staining (Figure 3c,d), LDH release (Figure 3e), and cardiac HMGB1 levels (Figure 3f) and release (Figure 3g). These results suggest that necroptosis is, at least in part, responsible for MI/R injury aggravation in aged hearts.

**Figure 4:**
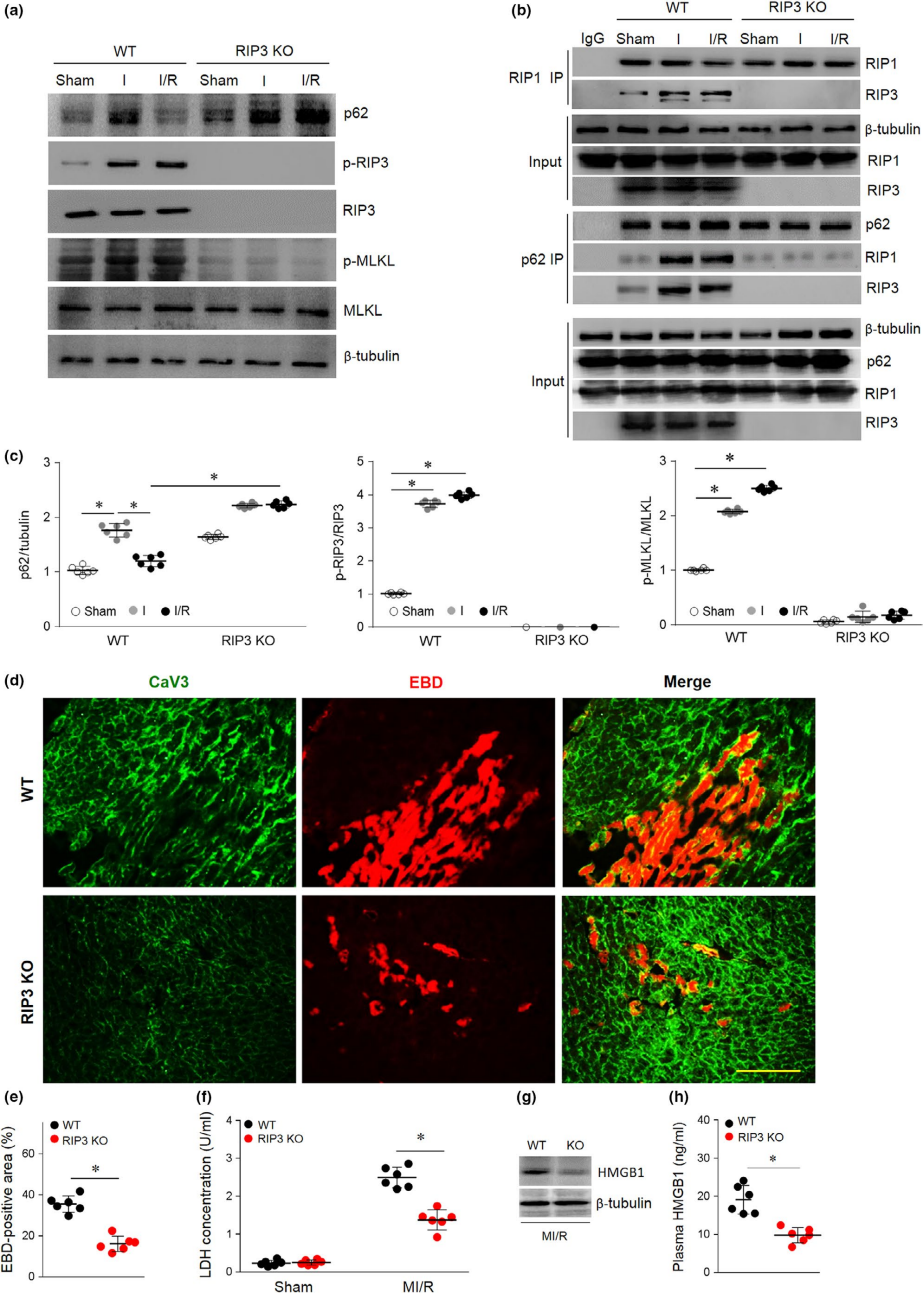
RIP3 is required for p62 accumulation‐enhanced myocardial necroptosis. (a) WT and RIP3 KO mice were subjected to 30 min cardiac ischemia and 4 hr reperfusion; then, the p62‐RIP1‐RIP3 complex and the RIP1‐RIP3 complex were examined by co‐immunoprecipitation. (b and c), Myocardial p62, p‐RIP3/RIP3, and p‐MLKL/MLKL were detected and quantitative by immunoblotting. (d and e) Myocardial necroptosis was evaluated by EBD uptake (red). (f) Serum LDH concentration in each group. (g and h) Representative Western blot and averaged data for cardiac or plasma HMGB1 levels in WT or RIP3 KO mice subjected to I/R injury. Scale bar = 20 μm. The values are the means ± *SEM*, *n* = 6 per group, *p < .05 versus the indicated groups

Because RIP3 is a key determinant of necroptosis in cardiac myocytes, we first investigated the potential role of RIP3 in linking p62 to necroptosis using RIP3‐deficient (RIP3 KO, 3–4 months) mice (Figure S3 and Table S2). Notably, although RIP3 KO hearts showed marked p62 accumulation during I/R (Figure 4a,b), RIP3 deficiency reduced p62‐RIP1‐RIP3 complex formation (Figure 4c) and blocked MI/R‐induced MLKL phosphorylation (Figure 4a,b). RIP3 KO mouse hearts were resistant to I/R‐induced myocardial necrosis, as evidenced by reductions in EBD penetration (Figure 4d,e), LDH release (Figure 4f) and cardiac HMGB1 levels (Figure 4g) and release (Figure 4h). Thus, the in vivo data indicate that RIP3 is required for p62‐induced myocardial necroptosis.

**Figure 5:**
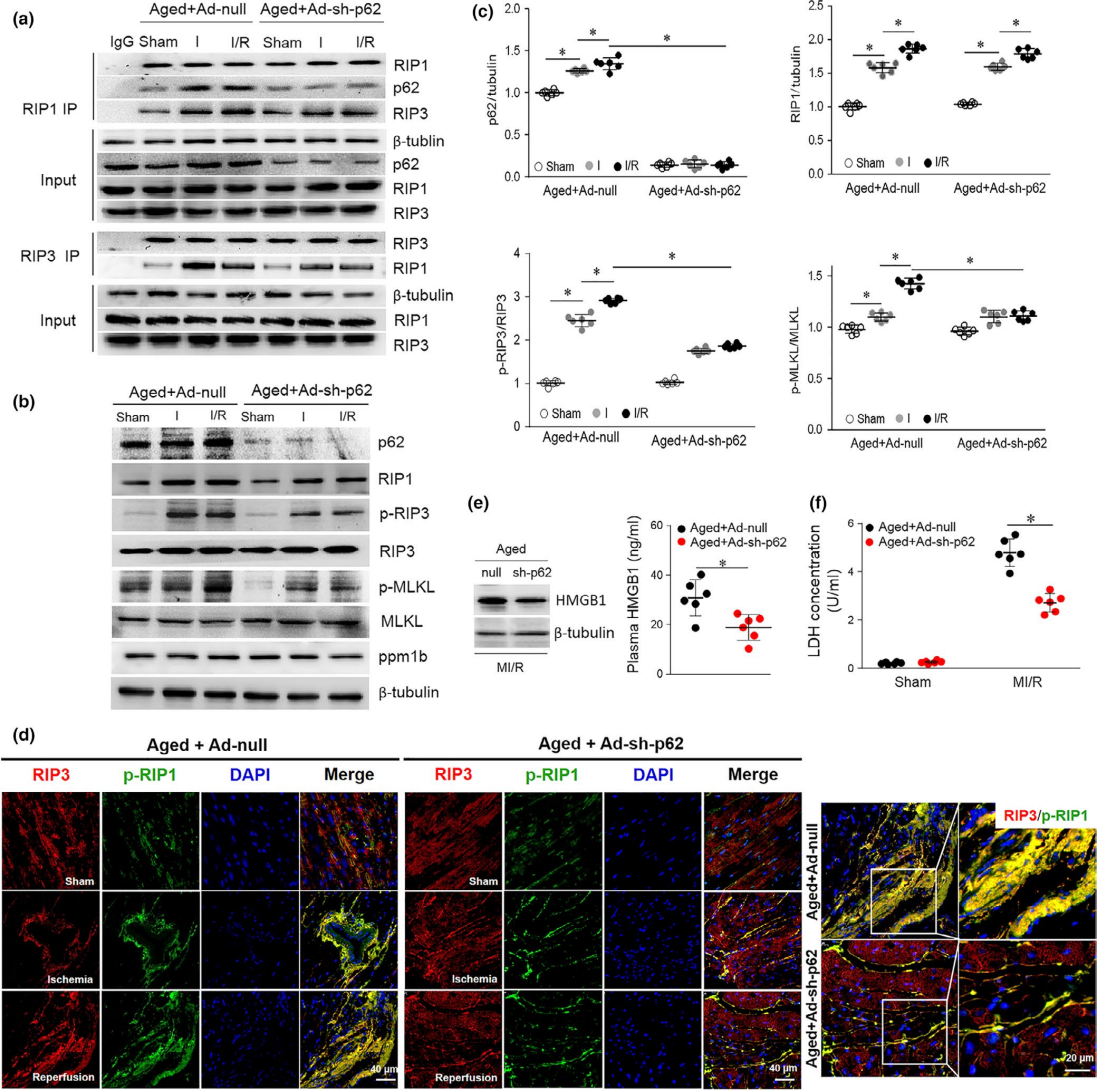
p62 knockdown in vivo reduces I/R‐induced necroptosis in aged hearts. (a) Representative co‐immunoprecipitation analysis of the p62‐RIP1‐RIP3 complex and the RIP1‐RIP3 complex in aged hearts treated with the adenovirus vector encoding p62‐shRNAs or scrambled shRNA. (b and c) Myocardial p62, p‐RIP3/RIP3, p‐MLKL/MLKL, and ppm1b were detected and quantitative by immunoblotting during I/R. (d) Representative photomicrographs for myocardial RIP3 (red) and p‐RIP1 (Ser166) (green) co‐localization staining by immunofluorescence. (e) Representative Western blot and averaged data for cardiac and plasma HMGB1 levels in aged mice subjected to I/R injury with or without p62 knockdown. (f) Serum LDH concentration in each group. Scale bar = 20 μm. The values are the means ± *SEM*, *n* = 6 per group, *p < .05 versus the indicated groups

Based on our observations, we hypothesized that p62 downregulation in aged hearts would reduce its accumulation and reduce its facilitation of necroptosis, thus reducing vulnerability to MI/R injury. For this purpose, aged mice were ventricularly injected with an adenovirus vector encoding a small hairpin RNA (shRNA) against the p62 gene (Ad‐sh‐p62) or Ad‐sh‐null as a control. Seventy‐two hours after infection, cardiac p62 levels were significantly decreased in aged hearts (Figure S4). p62 silencing blocked p62‐RIP1‐RIP3 binding and reduced necrosome formation (Figure 5a). p62 silencing did not affect RIP1, RIP3, PPM1B, or MLKL expression, while MI/R‐induced RIP3 and MLKL phosphorylation was notably decreased in the aged hearts (Figure 5b,c). We also determined that RIP1 and RIP3 were colocalized using coimmunofluorescence in p62‐silenced aged hearts. The fluorescent co‐localization of p‐RIP1 and RIP3 was significantly enhanced during MI/R in aged hearts (Figure 5d). However, p62 silencing eliminated p‐RIP1 and RIP3 co‐localization. Ultimately, p62 silencing in vivo protected the aged hearts from necrosis, as evidenced by reduced cardiac HMGB1 levels (Figure 5e), HMGB1 plasma release (Figure 5e), and LDH release (Figure 5f). These data suggest that in aged hearts, p62 may stabilize the RIP1‐RIP3 complex and that in vivo silencing of p62 inhibited RIP1‐RIP3‐induced necroptosis signals during MI/R conditions.

### 2.5 Metformin restores autophagy flux and decreases necroptosis

**Figure 6:**
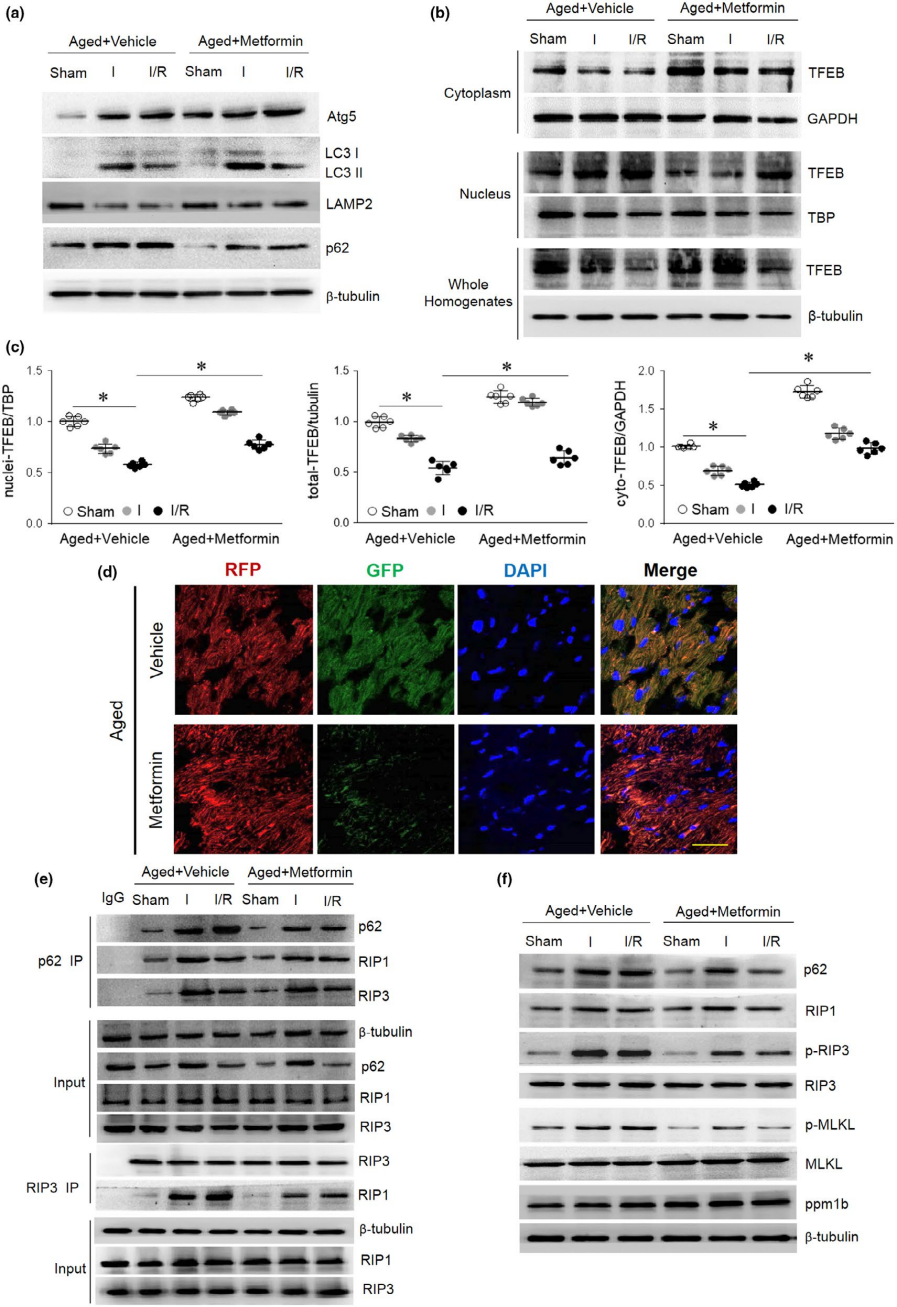
Metformin restores autophagy flux. Aged mice were injected intraperitoneally with metformin (125 μg/kg) or a vehicle control 4 weeks prior to I/R surgery. (a and b) TFEB expression in the cytoplasm, nuclei, and whole homogenates was detected by immunoblots. (c) Myocardial autophagic markers Atg5, LC3 II/I, LAMP2, and p62 were detected by immunoblotting. Quantification data are included in Figure S6. (d) Myocardial RFP‐GFP‐LC3 was stained and imaged by immunofluorescence in aged mice with or without metformin treatment. (e) Representative co‐immunoprecipitation analysis of the p62‐RIP1‐RIP3 complex and the RIP1‐RIP3 complex in aged hearts with or without metformin treatment. (f) Immunoblotting shows p62, RIP1, p‐RIP3/RIP3, p‐MLKL/MLKL, and ppm1b in each group. Quantification data are included in Figure S7. Scale bar = 20 μm. The values are the means ± *SEM*, *n* = 6 per group, *p < .05 versus the indicated groups

Our results suggest that aging‐associated cardiac necroptosis enhancement is due to autophagy inhibition. Thus, restoring autophagy may inhibit I/R‐induced necroptosis, protecting the aged heart. Metformin, a potential anti‐aging agent, is thought to accelerate autophagy (Song et al., 2016). However, the effect of metformin on autophagy‐necroptosis crosstalk in the aging myocardium is not yet known. Metformin (125 μg/kg, i.p.) or an equivalent volume of vehicle was administered to aged mice for 4 weeks (Sun & Yang, 2017) (Table S3), and the mice were subjected to MI/R injury. Compared with the vehicle control, metformin administration to aging mice markedly increased the myocardial phosphorylation of AMPK, whereas phosphor‐mTOR (p‐mTOR) levels decreased during ischemia (Figure S5). Furthermore, transcription factor EB (TFEB) is a master regulator of the autophagy–lysosome machinery and thus sustains autophagy flux (Lapierre, Kumsta, Sandri, Ballabio, & Hansen, 2015; Ma et al., 2015; Settembre et al., 2011). Confirmatory results were obtained by analyzing myocardial TFEB abundance in the nuclei, cytoplasm, and whole‐heart lysates from aged mice with or without metformin treatment and subjected to I/R injury. We found that in aged hearts, in vivo I/R injury suppressed TFEB levels and reduced nuclear TFEB abundance (active form) by 46% compared to the sham operation. Importantly, metformin treatment prevented the decrease in TFEB abundance and nuclear TFEB levels upon I/R injury in aged hearts (Figure 6a,b). These effects were associated with increased Atg5, LC3‐II, and LAMP2 levels, decreased p62 levels (Figure 6c, Figure S6) and autophagic flux stimulation, indicated by decreased GFP‐RFP‐LC3 (yellow fluorescence) (Figure 6d), in I/R‐injured aged hearts.

Simultaneously, metformin treatment resulted in an observed decrease in the p62‐RIP1‐RIP3 interaction as well as necrosome formation during I/R in aged hearts (Figure 6e). I/R‐induced p62 accumulation and necroptosis activation (p‐RIP3 and p‐MLKL) in aging hearts were lower in the metformin group than in the vehicle control group (Figure 6f, Figure S7). Immunofluorescence in vivo results showed that metformin treatment effectively prevented the I/R‐induced membrane translocation of p‐MLKL (Figure S8). Consistent with these biochemical data, we found that metformin treatment protects the aged heart from MI/R‐induced necrosis (as assessed by EBD‐positive staining and LDH release, Figure S9A,B), reduces cardiac contractile dysfunction (as indicated by increases in the ejection fraction and fractional shortening, Figure S9C), and reduces the myocardial infarct size (Figure S9D). Ultimately, metformin treatment significantly decreased mortality in I/R‐injured aged mice (Figure S9E).

## 3 DISCUSSION

In the present study, we demonstrated that metformin treatment repressed myocardial necroptosis in aging. Our in vivo results identified that aging facilitates RIP1‐RIP3 activation by p62 accumulation, which promotes myocardial I/R injury. Our findings show that in aged mice and human myocardium samples, autophagy is defective, as indicated by p62 accumulation. This mechanistic study demonstrated that p62 forms a complex with RIP1‐RIP3 in vivo and promotes the binding of RIP1 and RIP3, which sensitize cardiomyocytes to ischemic necroptosis. Metformin treatment restores autophagy flux in aged hearts and decreases p62 interaction with necrosomes in I/R‐injured myocardium, ultimately reducing mortality in this model. We conclude that p62 accumulation provides a direct link between aging and the onset of necroptosis enhancement in a pathophysiological context (Figure S9F).

Essentially, excessive death of terminally differentiated cardiomyocytes is an important pathogenic factor in aging‐related ischemic susceptibility (Adameova, Goncalvesova, Szobi, & Dhalla, 2016). The loss of cardiac myocytes has traditionally been believed to occur mainly due to programmed apoptosis or unregulated necrosis. Over the past decade, necroptosis has attracted the most attention, and its participation in cardiomyocyte loss has been studied in myocardial ischemic injury (Adameova et al., 2017; Zhang et al., 2016). However, whether necroptosis is involved in aging‐related myocardial ischemic intolerance has not been explored. Necroptosis is a tightly regulated process (Moe & Marin‐Garcia, 2016). The results of this study reveal the importance of RIP1‐RIP3‐evoked myocardial necroptosis in aging heart I/R injury. We found that necroptosis participates in aging‐related MI/R injury enhancement. In fact, I/R‐induced necroptosis in aging myocardium is significantly enhanced. Similar to the protective effects of RIP3 deficiency, Nec‐1 treatment markedly inhibited the RIP1‐RIP3 interaction in aged hearts and protected against I/R‐induced myocardial necrosis. Inhibiting necroptosis is an important aspect of cardioprotection in aged hearts during I/R injury, and the signaling mechanism underlying the aging‐related increase in necroptosis is located in the heart.

The relationship between autophagy and myocardial cell death has long been discussed. Cardiomyocytes are terminally differentiated, and protein aggregates and damaged intracellular organelles are not diluted through cell division. The abnormal events in the process of cell aging and the degradation of denatured proteins are performed by mainly the cardiomyocytes themselves. We believe that aging‐related myocardial autophagy dysfunction under the clinical threshold (subclinical myocardial aging) will seriously affect the clinical course and prognosis of cardiovascular disease. As a physiological process that effectively prevents the accumulation of abnormal proteins and organelles, autophagy is crucial for maintaining cell self‐renewal and energy homeostasis (Sciarretta, Maejima, Zablocki, & Sadoshima, 2018). Autophagy preserves cardiac structure and function under baseline conditions and is activated during stress (Sasaki, Ikeda, Iwabayashi, Akasaki, & Ohishi, 2017), limiting damage under most conditions (Sciarretta et al., 2018). Functional autophagy reduces injury and preserves cardiac function during ischemia (Godar et al., 2015; Sciarretta, Yee, Shenoy, Nagarajan, & Sadoshima, 2014). Otherwise, autophagy impairment is involved in the development of aging‐induced cardiac abnormalities (Eisenberg et al., 2016; Shirakabe et al., 2016). Consistent with these functions, autophagy constitutes a stress adaptation to avoid cardiomyocyte death, although massive autophagy activation may be detrimental for the heart under certain stress conditions. It is important that during the aging process, cardiac autophagy is progressively suppressed (Ren, Sowers, & Zhang, 2018; Shirakabe et al., 2016); thus, downregulating autophagy may underlie the progression of cardiac aging (Linton, Gurney, Sengstock, Mentzer, & Gottlieb, 2015; Ren et al., 2017). Age‐related pathologies and the aging process itself are accompanied by impaired protein quality control, dysfunctional protein accumulation, and proteostasis imbalance, all of which contribute to increased susceptibility to stress (Sciarretta et al., 2018). The over‐accumulation of autophagic machinery components can directly affect cell survival and death (Nah et al., 2016). The crosstalk between the autophagy machinery and apoptosis or necroptosis has gained increasing attention (Goodall et al., 2016). The molecular mechanism connecting autophagy decline with cardiac necroptosis in aging hearts is currently unknown. A recent study from Ogasawara et al. (2017) has shown that necroptosis activation suppresses autophagic flux and restoring autophagic flux protects cardiomyocytes from necroptosis in H9c2 cells (a permanent cell line derived from rat cardiac tissue; these cells do not possess heart‐specific morphological structures). However, the relationship between autophagy and necroptosis signaling pathways as well as the role of this intermodulation mechanism in preventing I/R injury in aged myocardium have not been systematically examined. Specifically, in the I/R in vivo model, we found that increased p62 accumulation and increased myocardial necrosis coexist in aging myocardium. We recently showed that p62 binds constitutively to RIP1 and RIP3 in vitro and in vivo, and this binding enhances the RIP1‐RIP3 complex. In addition, p62 knockdown weakens the formation of the RIP1‐RIP3 complex in aged mouse hearts. These data suggest that p62 accumulation induced by impaired autophagosome clearance contributes to sensitizing aging hearts to I/R‐induced myocardial necroptosis by interacting with the RIP1‐RIP3 complex. Our results establish a functional relationship between aging‐related cardiac autophagy dysfunction and necroptosis.

Metformin is a first‐line antidiabetic drug that also activates autophagy and has cardiovascular protective effects (Song et al., 2016; Teng et al., 2015), although recent study has reported that metformin fails to cardioprotective effect in nonaging I/R swine models (Techiryan et al., 2018). In the present study, we found that metformin treatment effectively reduced I/R‐induced necroptosis in aging hearts, improved cardiac function after myocardial infarction, and improved long‐term survival. Metformin treatment can restore autophagy and reduce p62 accumulation in aged myocardium, as well as decrease cardiac p62‐RIP1‐RIP3 binding, RIP1‐RIP3 complexes, and I/R‐induced RIP3 and MLKL phosphorylation. Metformin may break the unfavorable chain mechanism of aging‐related autophagy decreases that induce necroptosis, thus protecting aging hearts from MI/R injury. Our data extend the previous findings regarding the potential anti‐aging effects of metformin. Currently, clinical trials studying the therapeutic effects of metformin on longevity (ClinicalTrials.gov. Unique identifier: NCT02432287) are ongoing. It is tempting to speculate that our present observations may provide some new insight into these previously observed metformin‐mediated cardiac benefits.

In summary, our findings show that p62 accumulation is sufficient to promote necroptosis signals by the pro‐formation of the p62‐RIP1‐RIP3 complex, thereby enhancing MI/R‐induced necroptosis in aged hearts. Metformin ameliorates myocardial necroptosis induced by autophagic defects during cardiac aging. These findings confirm the new hypotheses regarding metformin‐mediated cardioprotection against aging‐induced ischemic necroptosis enhancement.

## 4 EXPERIMENTAL PROCEDURES

### 4.1 Animals and human samples

For details, see Appendix S2.

### 4.2 Antibodies and reagents

For details, see Appendix S2.

### 4.3 In vivo ischemia and reperfusion surgery

For details, see Appendix S2.

### 4.4 Cell culture and hypoxia/reoxygenation

For details, see Appendix S2.

### 4.5 Adenoviral transduction and fluorescence microscopy

For details, see Appendix S2.

### 4.6 Western blotting and co‐immunoprecipitation

For details, see Appendix S2.

### 4.7 Nuclear‐cytoplasmic protein extraction

The nuclear‐cytoplasmic extraction was performed according the protocol of the NE‐PER Nuclear and Cytoplasmic Extraction Reagents kit (No.78835, Thermo Fisher Scientific, USA) as previously described (Li et al., 2017).

### 4.8 In vitro GST pull‐down experiment

For details, see Appendix S2.

### 4.9 Histology and immunofluorescence

For details, see Appendix S2.

### 4.10 Immunohistochemistry

For details, see Appendix S2.

### 4.11 Adenovirus delivery

For details, see Appendix S2.

### 4.12 Cell membrane permeability to Evans blue dye

For details, see Appendix S2.

### 4.13 Lactate dehydrogenase (LDH) assay

For details, see Appendix S2.

### 4.14 Echocardiography

For details, see Appendix S2.

### 4.15 Statistical analysis

Data are expressed as mean ± *SEM*. Statistical analysis was performed with GraphPad PRISM version 5.01 (GraphPad Software, Inc.) and the SPSS 18.0 software package (SPSS Inc.). Data sets were tested for normality of distribution with Kolmogorov–Smirnov test. Data groups (two groups) with normal distribution were compared using two‐sided unpaired Student's *t* test. The Mann–Whitney *U* test was used for nonparametric data. Comparisons between multiple groups were assessed by one‐way ANOVA with Bonferroni post hoc analysis. *p < .05. No statistical method was used to predetermine sample size.

## CONFLICT OF INTEREST

None declared.

## AUTHOR CONTRIBUTIONS

Conception and design: Heng Ma and Lu Yu. Development of methodology: Heng Ma, Lu Yu, and Chen Li. Acquisition of data: Chen Li, Nan Mu, Zheng Yang, Yishi Wang, and Yue Yin. Analysis and interpretation of data: Zheng Yang, Manling Liu, and Mai Chen. Writing, review, and/or revision of the manuscript: Chen Li, Nan Mu, and Heng Ma. Administrative, technical, or material support: Chunhu Gu and Yuehu Han. Study supervision: Heng Ma and Lu Yu.

## Data Availability

Our article contains a Data Availability Statement, and additional supplemental data may be found online in the Supporting Information section at the end of the article.
