# Supplementary material for: Metformin mediates cardioprotection against aging‐induced ischemic necroptosis
Source: Aging Cell. 2020 Jan 14;19(2):e13096. doi: 10.1111/acel.13096 (PMC6996959; doi:10.1111/acel.13096)
Supplement: Supplementary file 2 [file ACEL-19-e13096-s002.docx]

**Supplementary Data**

**Title:** Metformin mediates cardioprotection against aging-induced ischemic necroptosis

Chen Li^1,^*, Nan Mu^1,^*, Chunhu Gu^2,^*, Manling Liu^1^, Zheng Yang^1^, Yue Yin^1^, Mai Chen^3^, Yishi Wang^1^, Yuehu Han^2^, Lu Yu^4,#^, Heng Ma^1,#^

^1^Department of Physiology and Pathophysiology; ^2^Department of Cardiovascular Surgery, Xijing Hospital; ^3^Department of Cardiovascular Medicine, Xijing Hospital;^4^Department of Pathology, Xijing Hospital; Fourth Military Medical University, Xi’an, 710032, China

* These authors contributed equally to this work

**Correspondence to:**

Heng Ma, M.D., Ph.D.

Department of Physiology and Pathophysiology, School of Basic Medical Sciences, Fourth Military Medical University, Xi’an 710032, China. Tel: 86-29-84774521, E-mail: hengma@fmmu.edu.cn;

or

Lu Yu, M.D., Ph.D.

Department of Pathology, Xijing Hospital, Fourth Military Medical University, Xi’an, 710032, China. E-mail: yulu@fmmu.edu.cn

**Supplementary Experimental Procedures**

***Animals and human samples***

Male C57BL/6 mice 3-4- (young) and 22-24-months-old (aging) were purchased from the animal center of the Fourth Military Medical University. RIP3^−/−^ (RIP3 KO, 3-4 monuths) mice were generated as previously described (Fan et al., 2016) (Supplementary Figure 2). The mice were assigned randomly to each experimental group. All animal experiments in this study were approved by the Animal Ethical Experimentation Committee of the Fourth Military Medical University. Aging mice were administered metformin (125 μg/kg, i.p.) for 4 weeks. The mice were provided adequate food and water and maintained under a 12-h dark-light cycle. Normal human myocardial tissues were from cardiac surgery patients from the Cardiac Surgery Department of Xijing Hospital of The Fourth Military Medical University. This experiment was approved by the Ethics Committee of The Fourth Military Medical University (QX20150611-2), and informed consent was obtained from all patients. Right ventricular outflow tract myocardium specimens (40–100 mg net weight) were resected during surgical repair. Subjects with high blood pressure, stroke, heart failure, hyperglycemia, dementia, abnormal hepatic and renal function and acute diseases were excluded from this study. Specimens were collected in sealed vials and stored at -80℃ until analysis.

Characteristic of normal human myocardial tissues (n=32) used in the study

| **Characteristic and Medication use** | **n=32** |
| --- | --- |
| Age (median, years) | 32 (7-70) |
| Sex, Female (%) | 50% |
| High blood pressure, positives (%) | 0 |
| Stroke, positives (%) | 0 |
| Heart failure, positives (%) | 0 |
| Hyperglycemia, positives (%) | 0 |
| Dementia, positives (%) | 0 |
| Abnormal hepatic/renal function, positives (%) | 0 |
| Other acute diseases, positives (%) | 0 |

***Antibodies and reagents***

Rabbit anti-Atg5 (1:1000), rabbit anti-LC3B (1:1000), rabbit anti-p62 (1:1000 for WB, 1:200 for IP), rabbit anti-LAMP2 (1:1000), rabbit anti-PPM1B (1:1000), rabbit anti-p-MLKL (1:1000), rabbit anti-caveolin-3 (1:1000), rabbit anti-ppm1b (1:1000), mouse anti-p62 (1:1000), mouse anti-RIP1 (1:1000) and secondary fluorescent antibodies (FITC, 1:3000; TRITC, 1:5000) were purchased from Abcam. Rabbit anti-RIP1 (1:1000 for WB, 1:200 for IP), rabbit anti-RIP3 (1:1000 for WB, 1:200 for IP), rabbit anti-p-RIP3 (1:1000), rabbit anti-MLKL (1:1000), rabbit anti-β-tubulin (1:1000) and HRP-linked secondary antibodies (1:5000) were purchased from Cell Signaling. Necrostatin-1 (Nec-1), metformin•HCl, rapamycin and bafilomycin A1 were obtained from Selleck. GST-Tag recombinant human RIP3 protein and His Tag recombinant human p62 were obtained from Abcam. Rabbit phospho-MLKL (Ser358) antibody (1:100) and rabbit phospho-RIP1 (Ser166) antibody (1:100) were obtained from Affinity.

***In vivo ischemia and reperfusion surgery***

Mice were anesthetized with 2% isoflurane, intubated, and ventilated with a rodent ventilator (Harvard Apparatus). A left lateral thoracotomy was performed, and the left anterior descending artery (LAD) was occluded with an 8-0 nylon suture for 30 min of ischemia, followed by 2 h (signaling evaluation) or 4 h (morphological analysis) of reperfusion. A polyethylene tube was placed below the ligature suture to prevent arterial injury. ECG confirmed the ischemic hallmark of ST-segment elevation during occlusion as previously described (Ma et al., 2010; Li et al., 2018; Ma, Guo, Yu, Zhang, & Ren, 2011). At different time points, the left ventricle (LV) was isolated and frozen in liquid nitrogen. Nec-1 was dissolved in PBS and injected (3.5 mg/kg, b.w.) into the mice via their tail vein 5 min prior to ischemia (Oerlemans et al., 2012). The hearts were excised and stained to determine the myocardial infarct size. Non-necrotic tissue was stained red with 1% 2,3,5-triphenyltetrazolium chloride (TTC), and the non-ischemic region was stained blue with 1% Evans blue dye. The infarct and left ventricular areas were photographed and measured by NIH ImageJ. The infarct size was calculated as the ratio of the infarct area to the area at risk (IF/AAR) (Zhang et al., 2016; Ma, Guo, Yu, Zhang, & Ren, 2011).

***Cell culture and hypoxia/reoxygenation***

Neonatal mouse ventricular myocytes were isolated from 1-day-old C57BL/6 mice. The animals were euthanized by cervical dislocation. Then, the hearts were obtained and digested in a digestion solution containing 0.25% trypsin and collagenase I. Myocytes were separated after 3 h of differential sedimentation and adhesion. The myocytes were then cultured in low glucose Dulbecco’s modified Eagle’s medium (DMEM) containing 10% fetal bovine serum (FBS). Rapamycin (100 nM in DMSO) and bafilomycin A1 (100 nM in DMSO) were added to the medium and incubated for 1 h before hypoxia. For the hypoxia experiments, the myocytes were continually cultured in N2/CO2 (95/5%) in an airtight incubator for 4 h and then reoxygenated for 4 h. The myocytes were digested with 0.25% trypsin and resuspended in PBS. The cells were stained with Annexin V-FITC/PI and detected by flow cytometry for cell death analysis.

***Adenoviral transduction and fluorescence microscopy***

The adenovirus mRFP-GFP-LC3 (Ad-tf-LC3) (Hanbio Inc, China) was transduced into neonatal mouse ventricular myocytes at 50 MOI for 24 h before drug treatment. After adenovirus transduction, the myocytes were fixed in 4% paraformaldehyde, incubated with 4’, 6-diamidino-2-phenylindole (DAPI) and viewed under a fluorescence microscope (Olympus Corporation, Japan). Autophagosomes and autolysosomes were counted according to the GFP and mRFP dots. DAPI-stained nuclei were counted to obtain the nuclei number. The dots/nuclei number was calculated by dividing the numbers from each microscopic field (Hariharan, Maejima, Nakae, Paik, Depinho, & Sadoshima, 2010).

***Western blotting and co-immunoprecipitation***

Western blotting was performed as previously reported (Wu et al., 2016). Co-immunoprecipitation was performed using a Pierce Co-IP Kit (No. 26149, Thermo Fisher Scientific). Briefly, the antibodies were first immobilized with coupling resin. Heart tissue was dissolved with IP-lysis buffer and pre-cleaned with control resin. The lysate was then mixed with immobilized resin overnight at 4°C. The binding proteins were eluted with elution buffer and prepared for western blotting analysis. Then, the proteins were visualized with an enhanced chemiluminescence detection kit (Millipore), and the results were analyzed with Quantity One software (BioRad Laboratories).

***In vitro GST pull-down experiment***

In vitro protein binding experiments were carried out according to the instructions of the GST Protein Interaction Pull-Down Kit (No. 78835, Thermo Fisher Scientific, USA). Briefly, glutathione agarose resins were added to the adsorption column and preconditioned with wash solution (TBS: pull-down lysis buffer, 1:1). GST fusion proteins were then added to the column, incubated with the resins on a gently shaking table for 1 h and washed with wash solution. The His-p62 fusion protein was added to the adsorption column and incubated with the resins for 1 h at 4℃. The resin and protein mixture was then washed with wash solution. The protein complexes were eluted with glutathione elution buffer and detected by western blotting.

***Histology and immunofluorescence***

Hearts were harvested, embedded in optimal cutting temperature (OCT) compound, snap frozen in liquid nitrogen-cooled isopentane and sliced into 20-µm-thick sections with a freezing microtome (Leica). The heart sections were fixed with 4% paraformaldehyde (PFA) and blocked with 10% serum in PBS. The sections were then incubated with the appropriate primary and secondary fluorescent antibodies and observed with a confocal fluorescence microscope (Olympus Corporation, Japan).

***Immunohistochemistry***

Animals were sacrificed and perfused with 4% paraformaldehyde. The hearts were removed, fixed in 4% paraformaldehyde, embedded in paraffin and sectioned. The sections were incubated sequentially in xylene I, II and III solutions for dewaxing and in 100%, 95% and 90% alcohol for dehydration. The sections were repaired with 0.1% sodium citrate buffer and incubated with H2O2 to block endogenous peroxidase. The sections were then incubated in a 0.03% Triton solution for 10 min and blocked with 10% donkey serum. Diluted p62 antibody was added to the sections (50 μL each) and incubated at 4°C overnight. An HRP and DAB solution was added sequentially for coloration. The sections were restained with hematoxylin and then treated with xylene for deparaffinization. All immunostained sections were observed and photographed under a confocal microscope (Olympus, Japan).

***Adenovirus delivery***

Adenoviruses encoding p62 shRNA (Ad-sh-p62) and adeno-associated virus 9 (AAV9)-mRFP-GFP-LC3 were purchased from Hanbio (Hanbio Corporation, Supplementary Table 4). Mice were intubated, ventilated and anesthetized with 2% isoflurane. A lateral sternotomy was performed at the level of the second intercostal. The heart was lifted from the thoracic cavity, and Ad-sh-p62 (1×10^11^ IFU/ml) and AAV9-mRFP-GFP-LC3 (1×10^12^ IFU/ml) (50 µl) were administered by directly injecting the LV from the apex using a 29-gauge insulin syringe needle. In addition, the aorta and pulmonary artery were clamped transiently with ophthalmic forceps for 30 s to send the virus into the coronary artery as previously described (Li et al., 2018). After virus injection, the residual air was evacuated with a 22-gauge plastic cannula, and the chest cavity was closed with a 5-0 Vicryl suture. The mice were then removed from the ventilator and allowed to recover. Myocardial p62 expression was analyzed 48 h later, and AAV9-mRFP-GFP-LC3 expression was observed 4 weeks after injection.

***Cell membrane permeability to Evans blue dye***

Myocardial necrosis was also assessed by measuring myocyte membrane permeability to Evans blue (Zhang et al., 2012). Evans blue dye was dissolved in saline (10 mg/ml) and injected into the mice (100 µg/g, bw, i.p.) 14 h before I/R surgery as previously reported (Zhang et al., 2012). The hearts were then harvested, frozen and sliced into 20-µm cryosections. Immunofluorescence was performed with an anti-CaV3 antibody, and the results were observed with confocal fluorescence microscopy.

***Lactate dehydrogenase (LDH) assay***

Blood samples were collected 24h after perfusion and centrifuged to obtain serum. Serum LDH was evaluated by Spectrophotometry as previously described (Xing, Sun, Wang, Gao, & Ma, 2016).

***Echocardiography***

Cardiac function was evaluated using a 2-D guided M-mode echocardiography. The mice were lightly anesthetized with 2 % isoflurane 24h after reperfusion. In vivo transthoracic echocardiography of left ventricle (LV) was used to obtain high-resolution two-dimensional mode images. LV ejection fraction (EF) were measured according to method of the American Society of Echocardiography.

**Supplementary Table 1.** **Gross and Echocardiographic** **features from young and aged mice**

| Mouse Group | Young | Aged |
| --- | --- | --- |
| Body weight(BW, g) | 25.96 ± 1.25 | 34.44±1.48* |
| Heart weight (mg) | 124.35 ± 5.13 | 170.75 ±7.52* |
| Tibial length (mm) | 17.12 ± 0.28 | 17.19±0.23 |
| HW/TL (mg/mm)  Heart rate (bpm)  LVIDd (mm)  LVIDs (mm)  FS (%) | 7.27 ± 0.33  441 ±17  3.85±0.13  2.54±0.10  33.91±4.27 | 9.93 ± 0.40*  433 ±14  3.93±0.14  2.57±0.13  34.58±3.96 |

Values are mean ± SEM; *n* = 20, **P*<0.05 *vs.* young , Student’s *t* test. HW, heart weight; TL, tibial length; LVIDd, left ventricle internal diameter diastolic; LVIDs, left ventricle internal diameter systolic; FS, fraction shortening.


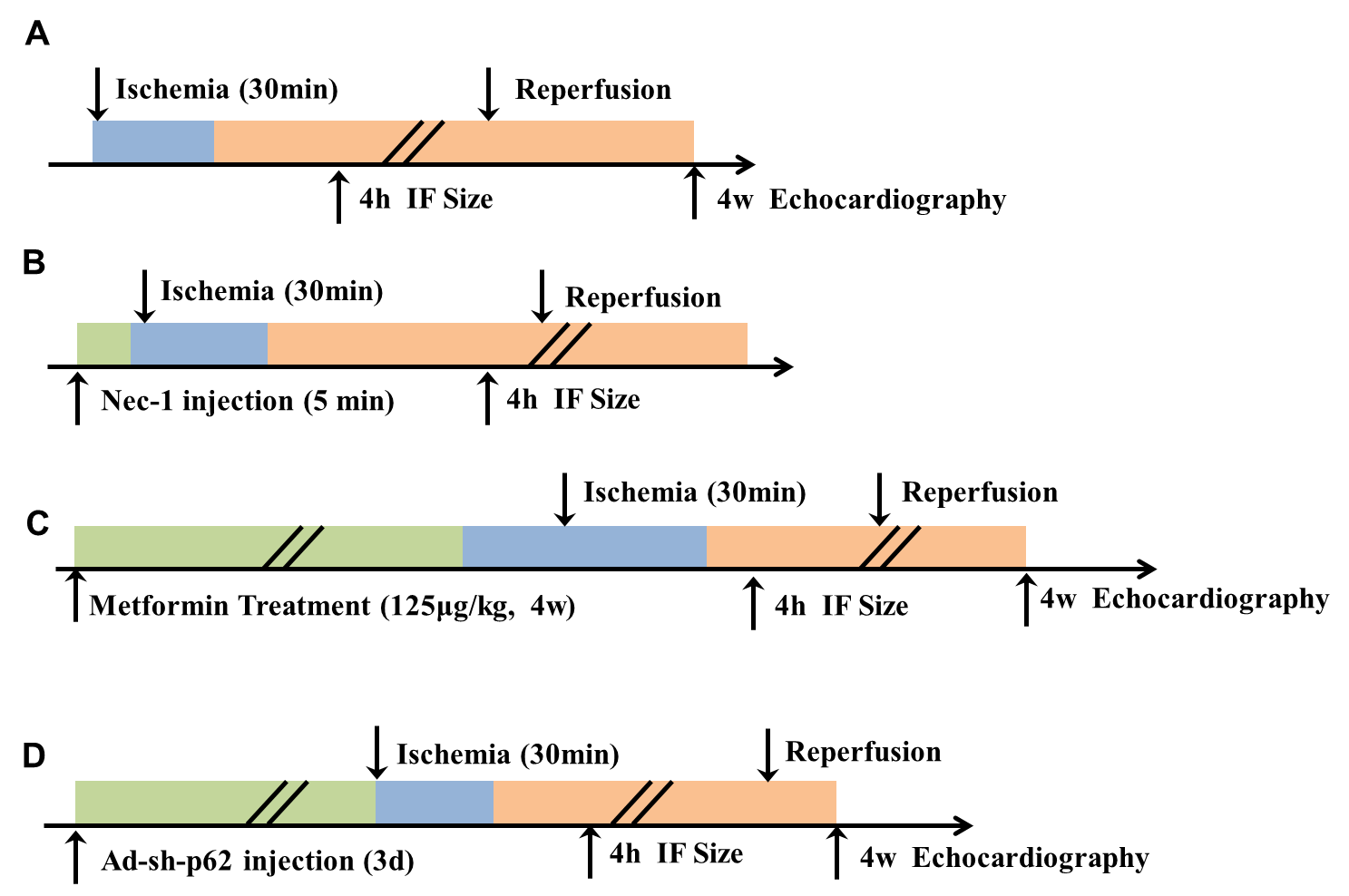


**Supplementary Figure 1. Experimental protocols *in vivo* diagrams.**

**A**. *In vivo* MI/R models (30-min ischemia and 4-h or 8-week reperfusion, respectively). **B**. Acute Nec-1 treatment of mice (3.5mg/kg, bw, 5 min prior to ischemia). **C**. Chronic metformin treatment of mice (125μg/kg, i.p. daily for 4 weeks). **D**. Ad-sh-p62 myocardial injection of mice (3 days prior to ischemia).


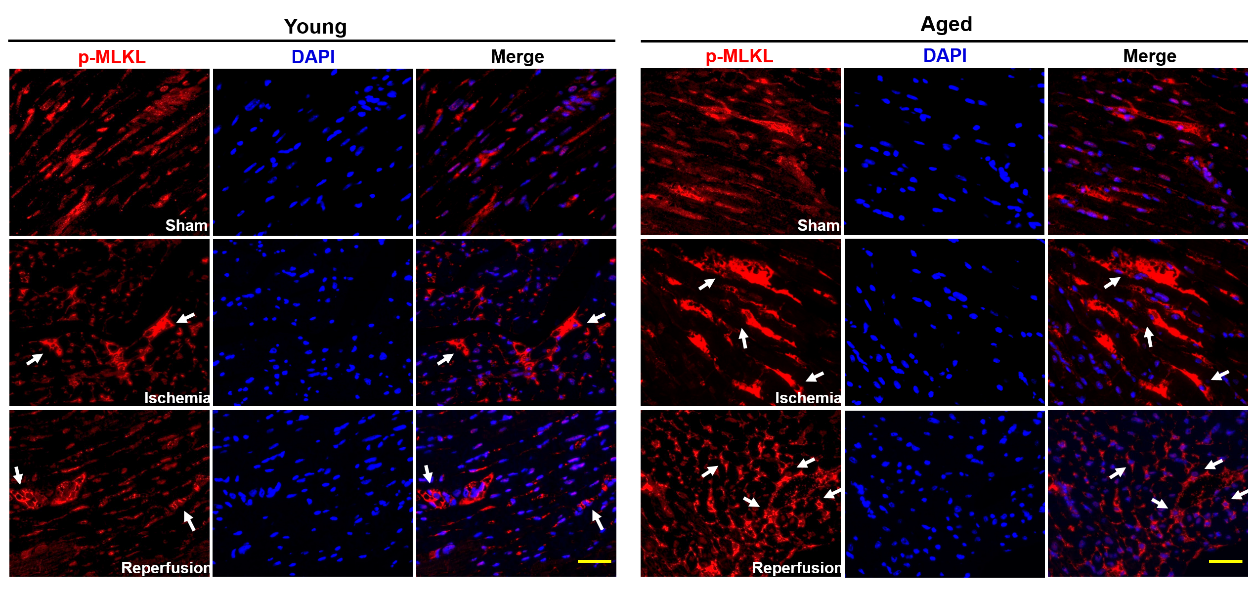


**Supplementary Figure 2. Representative photomicrographs for p-MLKL (Ser358) plasma membrane translocation during I/R as assessed by immunofluorescence in young and aged hearts.** Scale bar=40 μm.

**
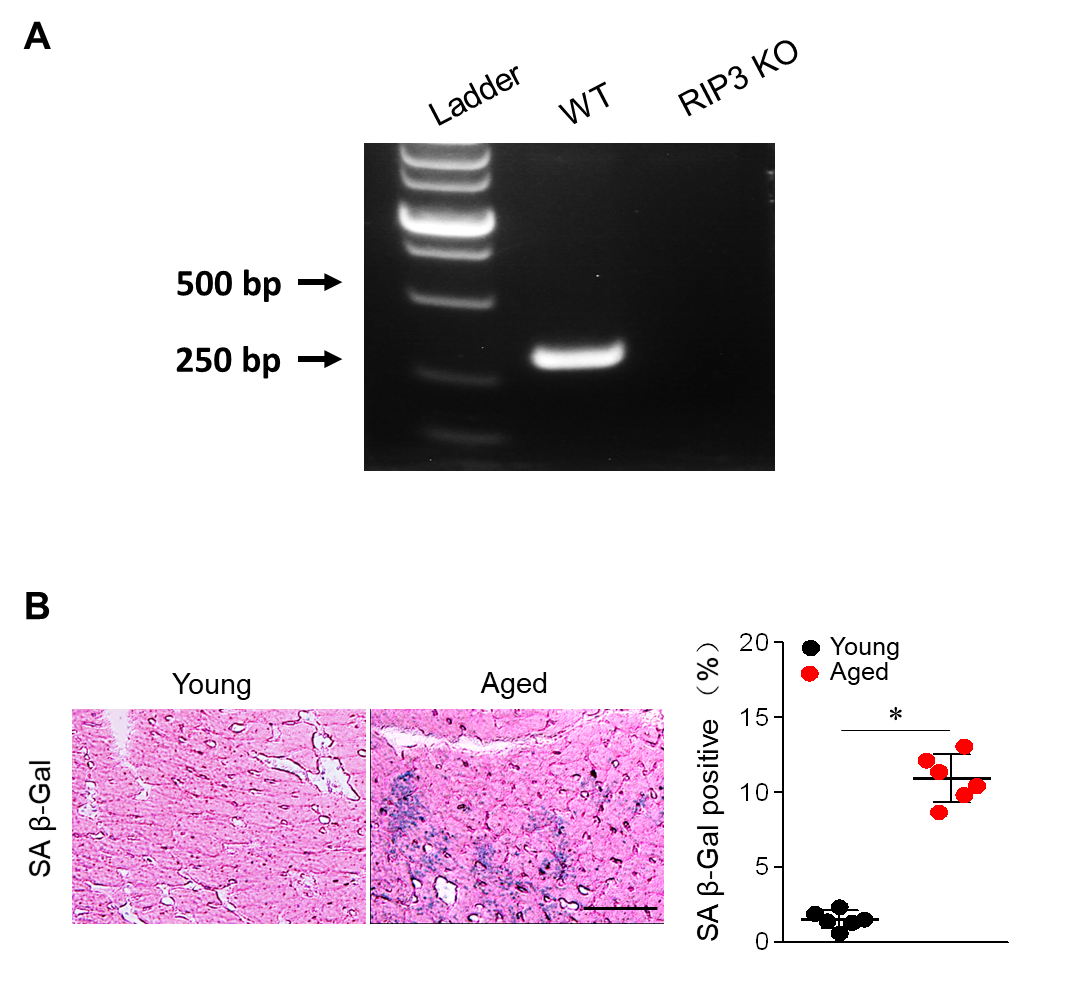
**

**Supplementary Figure 3. Genotyping identification of RIP3 KO mice and Histological staining of SA β-Gal.**

**A**. PCR genotyping of WT and Ripk3 KO mice. **B**. Histological staining of SA β-Gal in young and aged mouse hearts. Scale bar=20 μm. Values are means ± SEM, n=6 per group, **P*<0.05 versus as indicated.

**Supplementary Table 2. Gross and Echocardiographic features from WT and RIP3 KO mice**

| RIP3 KO Mouse Group | WT | RIP3 KO |
| --- | --- | --- |
| Body weight(BW, g) | 25.96 ± 1.25 | 24.23±1.30 |
| Heart weight (mg) | 124.35 ± 5.13 | 121.00±4.22 |
| Tibial length (mm) | 17.12 ± 0.28 | 17.04±0.14 |
| HW/TL (mg/mm)  Heart rate (bpm)  LVIDd (mm)  LVIDs (mm)  FS (%) | 7.27 ± 0.33  441 ±17  3.85±0.13  2.54±0.10  33.91±4.27 | 7.10±0.16  447±16  3.85±0.06  2.60±0.06  33.32±3.51 |

Values are mean ± SEM; *n* = 10, Student’s *t* test. HW, heart weight; TL, tibial length; LVIDd, left ventricle internal diameter diastolic; LVIDs, left ventricle internal diameter systolic; FS, fraction shortening.


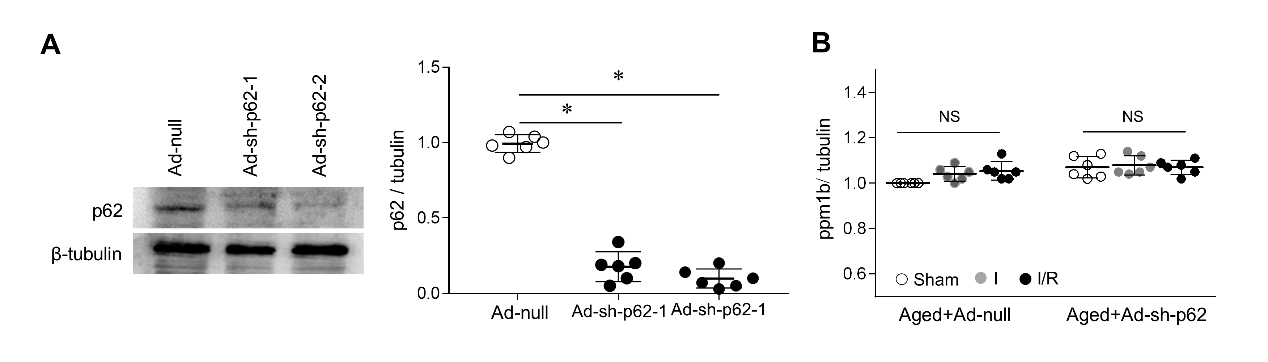


**Supplementary Figure 4. p62 knockdown *in vivo* in aged hearts.**

**A.** Representative western-blot analysis of myocardial p62 in aged hearts treated with the adenovirus vector encoding p62-shRNAs or scrambled shRNA. **B.** Myocardial ppm1b were detected and quantitative by immunoblotting during I/R in aged hearts treated with the adenovirus vector encoding p62-shRNAs or scrambled shRNA. The values are the means ± SEM, n=6 per group, **P*<0.05 versus the indicated groups.

**Supplementary Table 3. Gross and Echocardiographic from vehicle and metformin treated aged mice**

| Mouse Group | Vehicle | Metformin |
| --- | --- | --- |
| Body weight(BW, g) | 33.89 ± 1.51 | 30.23±1.86 |
| Heart weight (mg) | 169.65 ± 7.49 | 159.85 ±8.89 |
| Tibial length (mm) | 17.11± 0.18 | 17.09± 0.20 |
| HW/TL (mg/mm)  Heart rate (bpm)  LVIDd (mm)  LVIDs (mm)  FS (%) | 9.91±0.42  444 ±17  3.91±0.12  2.56±0.12  34.42±3.61 | 9.36±0.53  442±20  3.85±0.11  2.52±0.08  34.66±3.12 |

Values are mean ± SEM; *n* = 20, Student’s *t* test. HW, heart weight; TL, tibial length; LVIDd, left ventricle internal diameter diastolic; LVIDs, left ventricle internal diameter systolic; FS, fraction shortening.


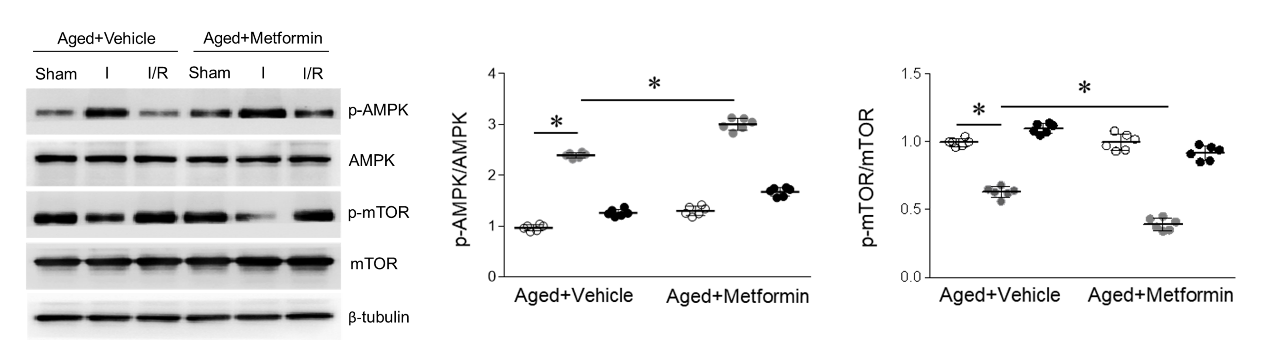


**Supplementary Figure 5. Metformin activates autophagy related signals in aging myocardium.**

Aged mice were injected intraperitoneally with metformin (125 μg/kg) or a vehicle control 4 weeks prior to I/R surgery. Myocardial p-AMPK/AMPK and p-mTOR/mTOR were detected by immunoblots. values are the means ± SEM, n=6 per group, **P*<0.05 versus the indicated groups.


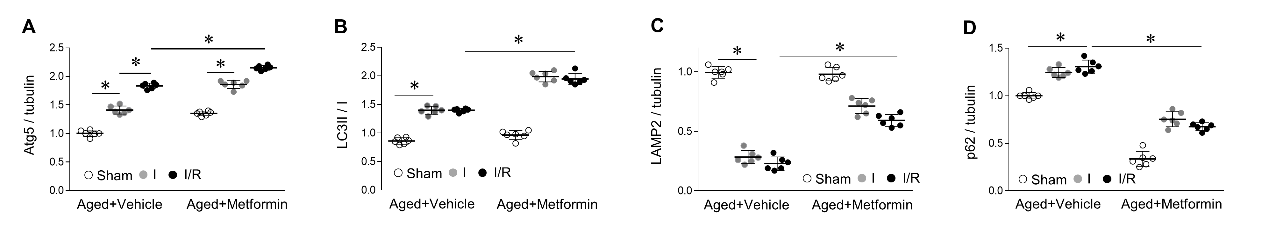


**Supplementary Figure 6. Quantitation of the Metformin activated autophagic markers in aging myocardium.**

Myocardial autophagic markers Atg5, LC3 Ⅱ/Ⅰ, LAMP2, and p62 were detected and quantitative by immunoblotting. Tubulin was used as a loading control. Values are means ± SEM, n=6 per group, **P*<0.05 versus as indicated.


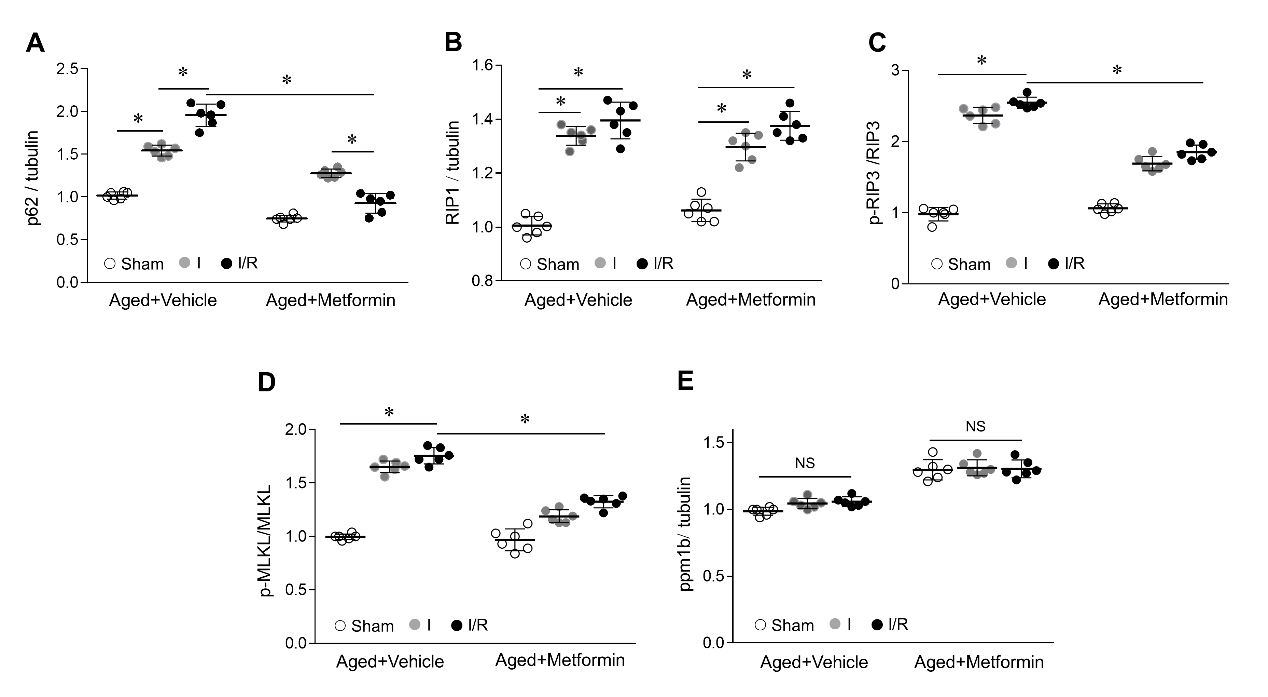


**Supplementary Figure 7. Quantitation of the p62 RIP1, p-RIP3/RIP3, p-MLKL/MLKL and ppm1b levels in Metformin treated aging hearts.**

The levels of p62 (A), RIP1 (B), p-RIP3/RIP3 ratio (C), p-MLKL/MLKL ratio (D) and ppm1b (E) were analyzed respectively. Tubulin was used as a loading control. Values are means ± SEM, n=6 per group, **P*<0.05 versus as indicated.

**
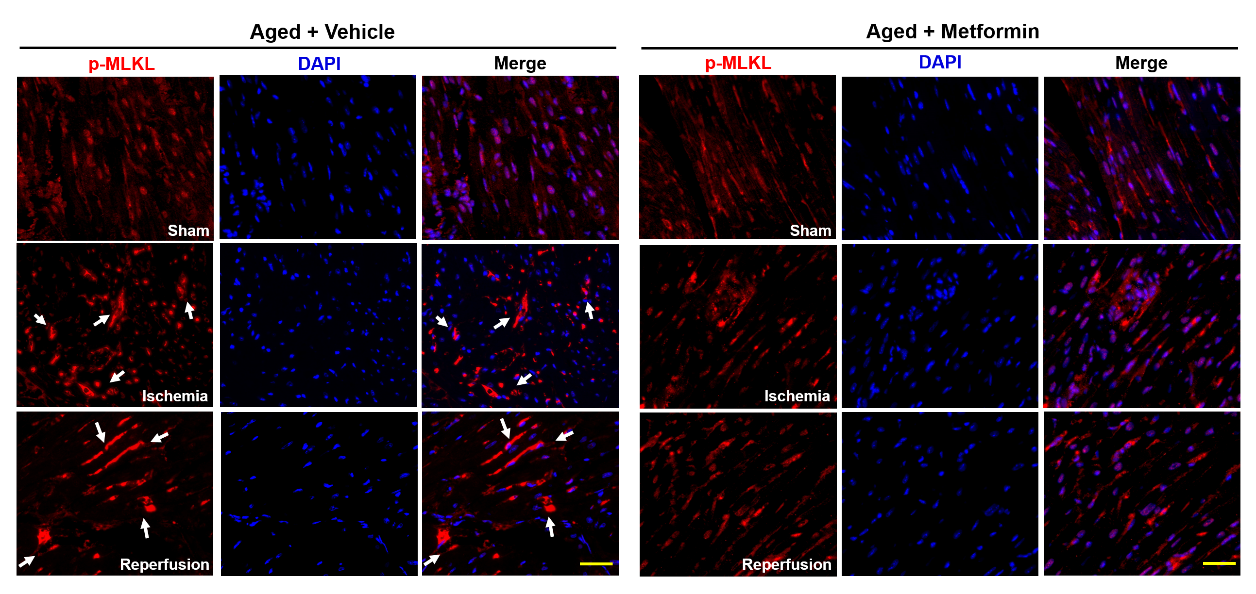
**

**Supplementary Figure 8. Representative photomicrographs for p-MLKL (Ser358) plasma membrane translocation during I/R as assessed by immunofluorescence in aged hearts with or without metformin treatment.** Scale bar=40 μm.


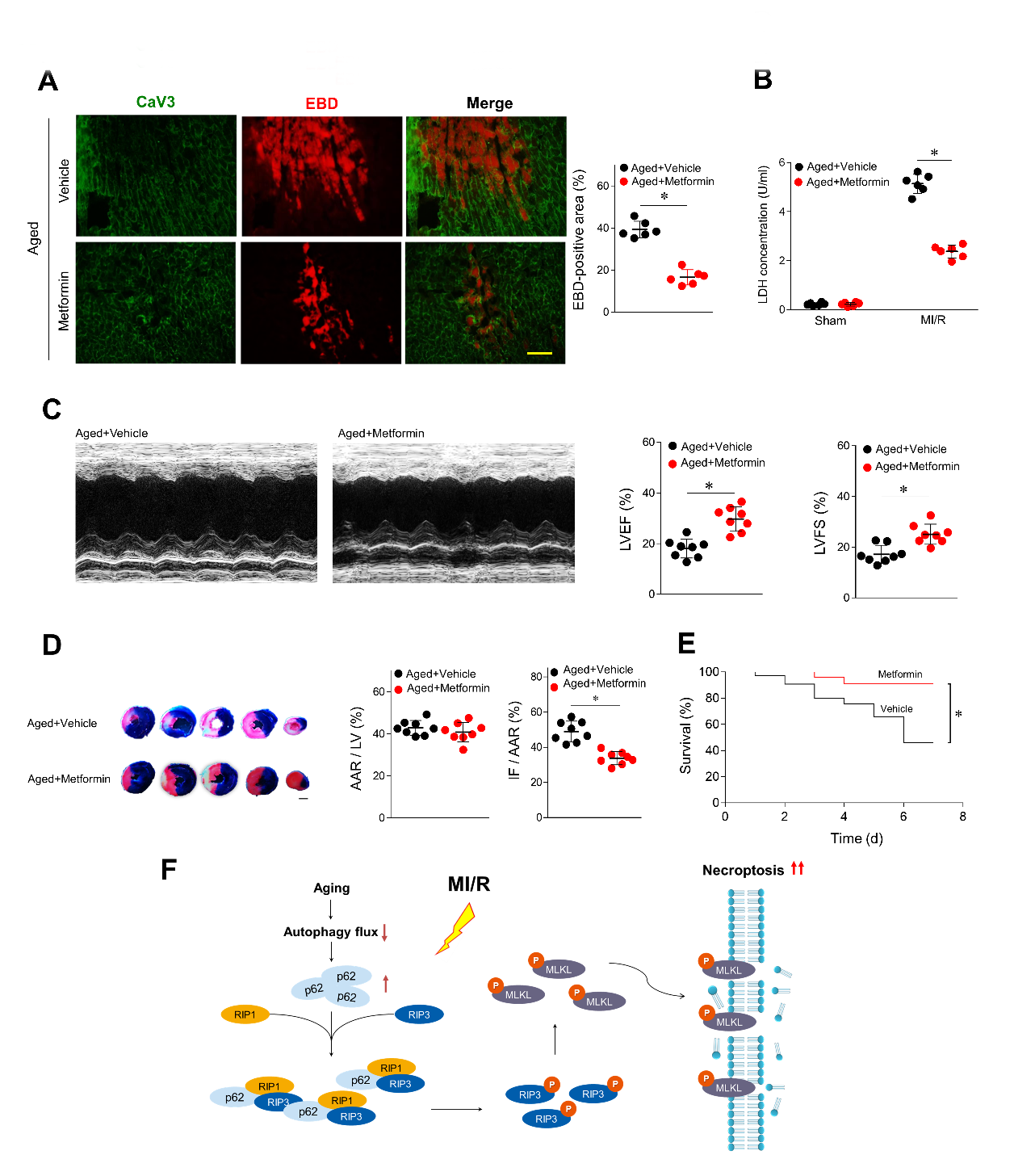


**Supplementary Figure 9. Metformin protects aged hearts from I/R-induced necrosis and heart failure.**

Aged mice were injected intraperitoneally with metformin (125 μg/kg) or a vehicle control 4 weeks prior to I/R surgery (30 min of ischemia and 4 h of reperfusion). **A**. Representative photomicrographs (left) and averaged data (right) from the images of myocardial EBD uptake and viable cardiomyocytes labeled with caveolin 3 (CaV3) antibody in aged mice subjected to I/R injury with or without metformin treatment. Scale bar=20 μm. **B**. Serum LDH concentration in each group. **C**. Representative photomicrographs and averaged data for the ejection fraction (EF) and fractional shortening (FS) assessed by echocardiography. **D**. Representative photographs and quantitative data for infarct size (IF) and area at risk (AAR) in aged hearts. Scale bar=2 mm. The values are the means ± SEM, n=6 per group, **P*<0.05 versus the indicated groups. **E**, Survival curve of aged mice subjected to I/R with or without metformin treatment, n=20, **P*<0.05 versus the indicated groups. **F**, Summary of working hypothesis: p62 accumulates and interacts with the RIP1-RIP3 complex to promote I/R-induced myocardial necroptosis in aged hearts.

**Supplementary Table 4. The Ad-sh-p62 and AAV9-GFP-RFP-LC3 sequences.**

| Gene | Gene ID | sequence |
| --- | --- | --- |
| Sqstm1/p62 | NM_011018.3 | AATTCGTGTGGTGGGAACTCGCTATAATTCAAGAGATTATAGCGAGTTCCCACCACATTTTTTG |
| Sqstm1/p62 | NM_011018.2 | CCGGTGTGGTGGGAACTCGCTATAACTCGAGTTATAGCGAGTTCCCACCACATTTTTG |
| MAP1LC3A | NM_032514.3 | ATGCCGTCGGAGAAGACCTTCAAGCAGCGCCGCACCTTCGAACAAAGAGTAGAAGATGTCCGACTTATTCGAGAGCAGCATCCAACCAAAATCCCGGTGATAATAGAACGATACAAGGGTGAGAAGCAGCTTCCTGTTCTGGATAAAACAAAGTTCCTTGTACCTGACCATGTCAACATGAGTGAGCTCATCAAGATAATTAGAAGGCGCTTACAGCTCAATGCTAATCAGGCCTTCTTCCTGTTGGTGAACGGACACAGCATGGTCAGCGTCTCCACACCAATCTCAGAGGTGTATGAGAGTGAGAAAGATGAAGATGGATTCCTGTACATGGTCTATGCCTCCCAGGAGACGTTCGGGATGAAATTGTCAGTGTAA |
