# Supplementary material for: Metformin mediates cardioprotection against aging‐induced ischemic necroptosis
Source: Aging Cell. 2020 Jan 14;19(2):e13096. doi: 10.1111/acel.13096 (PMC6996959; doi:10.1111/acel.13096)
Supplement: Supplementary file 1 [file ACEL-19-e13096-s001.docx]

**Response to Reviewers’ comments**

Dear Editor and Reviewers,

We really appreciate your evaluating of our research work reported in this manuscript with care and dispatch. We have read the comments carefully, and performed some additional experiments, especially the p-RIP1/RIP3 and p-MLKL immunofluorescence staining, according to your suggestions. We now make a point-by-point response to your comments and questions that you have concerned.

**Answers to reviewers # 1:**

**Reviewer: 1**

**Comments to the Author**

**(There are no comments.)**

**Answers to reviewers # 2:**

**Reviewer: 2**

**Comments to the Author**

**The authors have addressed my concerns in a fine manner.**

**Answers to reviewers # 3:**

**Reviewer #3:**

**Comments to the Author**

**I fully understand that analysis for animal model to explain the mechanism is very difficult. However, some of approach should make solid results to make their study better. One example, the authors mentioned that “there are no appropriate antibodies on the market to detect phospho-RIP1 or phospho-RIP3 by immunofluorescence staining. So we used RIP1 and RIP3 antibodies instead to perform immunofluorescence staining and the co-localization of RIP1 and RIP3 represents the combination of RIP1/RIP3 partially.”. This statement is not suitable for the response to my point. As I mentioned that these two proteins are expressed in cytosol. So if you stained cells with RIP1 antibody, most of cytosol are positive signal without any activation. It is same in RIP3. I think the authors understand this situation but mentioned that “although the co-location results are flawed, our results can support the conclusions of this study”. I could not agree with this. Most of the response to my points are not fully addressed and not satisfied.**

**Answer:** We are very grateful to reviewer for your meticulous work and positive comments. According to the concerns which you have mentioned, we have performed some additional experiments, especially the p-RIP1/RIP3 and p-MLKL immunofluorescence staining.

① p-RIP1 and RIP3 immunofluorescence staining

As we have mentioned in the firstly revised manuscript, the phospho-RIP1 (Ser166) antibody (Affinity, AF2398), was used in immunofluorescence staining. To improve the staining approach, paraffin-embedded heart specimens were cut on a microtome into 5-μm sections, and as showing in Figure 5D (revised), the myocardial fluorescent co-localization (yellow) of p-RIP1 (Ser166) (green)and RIP3 (red) was significantly enhanced during MI/R in aged hearts. However, p62 silencing eliminated p-RIP1 and RIP3 co-localization.

② p-MLKL immunofluorescence staining.

We performed immunofluorescence staining to verify the MLKL translocation as adjunctive evidence. Similarly, we used paraffin-embedded heart specimens were cut on a microtome into 5-μm sections, and phospho-MLKL (Ser358) antibody (Affinity, AF7420) to perform immunofluorescence staining and the localization of p-MLKL represents plasma membrane translocation. As showing in Supplementary Figure 2 (revised) and Supplementary Figure 8 (revised), I/R distinctly increased p-MLKL (red) membrane translocation, and metformin treatment effectively prevented the I/R-induced membrane translocation of p-MLKL. Thank you again for your advice to make our study better.

**Supplementary Figure 2.** Representative photomicrographs for p-MLKL (Ser358) plasma membrane translocation during I/R as assessed by immunofluorescence in young and aged hearts. Scale bar=40 μm.

**Supplementary Figure 8.** Representative photomicrographs for p-MLKL (Ser358) plasma membrane translocation during I/R as assessed by immunofluorescence in aged hearts with or without metformin treatment. Scale bar=40 μm.
